# Exploring the profile and risk factors associated with self-harm ideation and behaviors in adolescents with high psychoticism

**DOI:** 10.3389/fpsyt.2025.1555780

**Published:** 2025-03-24

**Authors:** Xiao-Ming Xu, Ru-Hong Jiang, Yu-Shuang Han, Wo Wang, Ming Ai, Jian-Mei Chen, Jun Cao, Xiao-Rong Chen, Zhen Lv, He-Yan Xu, Da-Qin Ding, Su Hong, Jing-Lan He, Qi Zhang, Lei Shi, Ning Du, Jin-Hui Hu, Li Kuang

**Affiliations:** ^1^ Department of Psychiatry, The First Affiliated Hospital of Chongqing Medical University, Chongqing, China; ^2^ Mental Health Center, University-Town Hospital of Chongqing Medical University, Chongqing, China; ^3^ Psychiatric Center, The First Affiliated Hospital of Chongqing Medical University, Chongqing, China

**Keywords:** adolescent, psychoticism, self-harm, suicidal ideation, suicide attempt, non-suicidal self-injury

## Abstract

**Background:**

As a core personality trait closely linked to mental health outcomes, psychoticism warrants particular attention in adolescent populations. The association between elevated psychoticism levels and self-harm ideation and behaviors (SIB) remained insufficiently characterized, especially regarding specific risk profiles. This study aims to delineate SIB characteristics and identify risk factors among adolescents with high psychoticisme.

**Methods:**

In this large scale cross-sectional study, 6,027 adolescents aged 16-18 years scoring ≥70th percentile on the psychoticism dimension (Revised Short Form of the Eysenck Personality Questionnaire, EPQ-RSC) were recruited from 63 schools. Data on demographic characteristics and SIB patterns were collected via standardized electronic questionnaires through a secure online platform. Binary logistic regression analyses with adjusted odds ratios (aOR) identified significant SIB predictors.

**Results:**

Adolescents with high psychoticism demonstrated striking SIB prevalence patterns: 62.8% reported life meaninglessness, 47.2% expressed death wishes, and 34.7% acknowledged suicidal/NSSI ideation within the past year, with 27% specifically endorsing such ideation within the past month. Actual self-harm rates were 4.7% (lifetime), 1.64% (past year), and 0.37% (past month). Predominant triggers included family conflicts (32.9%), while primary motives centered on pain alleviation (51%). SIB incidence showed significant dose-response relationship with psychoticism severity (*p<*0.05). Rural residency (aOR=1.32, 95% CI 1.04-1.675) and typical high psychoticism (aOR=1.509, 95% CI 1.16-1.963) emerged as key risk factors. Increasing age conferred significant protection against self-harm ideation (aOR=0.687, 95% CI 0.627-0.753), whereas female sex demonstrated differential risk modulation patterns across SIB (lifetime self-harm behavior aOR=2.053 vs. past-month self-harm ideation aOR=0.648).

**Conclusion:**

Our findings highlight the critical need for targeted interventions addressing modifiable determinants. Prioritizing female adolescents and those with higher psychoticism traits is recommended, supported by evidence-based family psychoeducation programs and enhanced accessibility of community mental health services with specific focus on emotion regulation training.

## 1 Introduction

Psychoticism, a core dimension in Eysenck’s tripartite personality model alongside neuroticism and extraversion-introversion (1), characterizes individuals through maladaptive traits including impulsivity, sensation-seeking, and interpersonal manipulation (2). Substantial evidence links this construct to mental health vulnerabilities (3, 4), with emerging longitudinal studies specifically implicating elevated psychoticism as a prognostic marker for adolescent self-harm ideation and behaviors (SIB). Meta-analytic data reveal moderate correlations between psychoticism and self-harm behaviors in collegiate populations (5), while prospective cohort studies demonstrate its predictive validity for adolescent self-harm behaviors trajectories (6). Lu et al.’s machine learning analysis of 60,000 Chinese undergraduates further has identified psychoticism as a significant factor in predicting suicidal ideation, suicide plan, and suicide attempt, especially in predicting suicidal ideation, where it showed the most pronounced effect (Shapley Additive Explanations value of 0.39) (7). Our previous work employing ensemble learning algorithms similarly identified psychoticism as the second and fifth most salient predictor in lifetime and past-year self-harm behavior risk models, respectively (8). Emerging evidence link elevated psychoticism to suicidality through empirically validated mediators, particularly psychache and perceived social disconnectedness (5, 9), though causal inference remains constrained by residual confounding in these observational studies.

Elevated psychoticism scores are observed not only in individuals with SIB but also among college students exhibiting internet addiction behaviors (10), patients diagnosed with antisocial personality disorder or bipolar disorder (11), and individuals with schizophrenia spectrum disorders and their first-degree relatives (12, 13). The broad association between psychoticism and diverse psychopathological manifestations limits its specificity as a diagnostic marker for particular mental disorders, while potentially explaining the lower measurement consistency of the psychoticism (P) factor compared with other EPQ dimensions (14, 15). Notably, emerging evidence indicates pathological P-score elevations in specific clinical populations. Pop-Jordanova et al. documented near-maximum P-scale raw scores (mean 16/18) in males with anorexia nervosa (16). Similarly, 17.1% of panic disorder patients exhibited converted P-scores ≥ 61.5 T-points, demonstrating significant divergence from controls (*p*<0.01) (17). Clinically, elevated P-scores may contraindicate endoscopic thoracic sympathectomy due to their strong correlation with postoperative compensatory hyperhidrosis (18). These findings collectively suggest that extreme P-score ranges may retain predictive utility for specific psychopathological outcomes. Nevertheless, given the multidimensional heterogeneity of psychoticism, this study proposed that differential P-score thresholds may correlate with distinct phenotypic dimensions of adolescent SIB, potentially enabling stratified risk prediction.

Although psychoticism is widely considered associated with SIB in adolescents, the specific features of SIB in adolescents with elevated psychoticism remain unclear. This study aims to (1) delineate the comprehensive characteristics of SIB in adolescents with high psychoticism levels, (2) elucidate the relationship between psychoticism severity and SIB manifestations, and (3) assess the impact of socio-demographic covariates. These objectives will promote a facet-level understanding of clinical symptomatology and establish a foundation for investigating psychoticism-related traits. By enhancing our comprehension of psychological states in self-harming adolescents, this research ultimately aims to inform the development of targeted prevention and intervention strategies.

## 2 Materials and methods

### 2.1 Participants

This secondary analysis utilized psychological screening data from a large-scale cross-sectional study conducted among Chongqing students between 2012 and 2015. A stratified cluster sampling design was employed, with school type (university, vocational college, or high school) serving as the stratification variable. The sampling framework included 63 institutions proportionally representing three educational tiers: 13 universities, 21 vocational colleges, and 29 high schools.

Within each institution, first-year classes were randomly selected to achieve a representative sample comprising 30% of the incoming student population. This yielded a final cohort of 136,266 participants who completed standardized questionnaires. Detailed methodological procedures for data collection and screening have been previously published (10, 19).

Adolescents aged 16-18 years with elevated psychoticism traits were identified using the Eysenck Personality Questionnaire-Revised Short Scale for Chinese (EPQ-RSC), with classification thresholds defined per established protocols (20). The study protocol received ethical approval from the Institutional Review Board of Chongqing Medical University. Prior to participation, all students received written disclosures regarding study objectives, data confidentiality protections, and voluntary participation principles. Implied consent was obtained through questionnaire completion.

### 2.2 Measures

The study employed a centralized online testing protocol implemented across participating schools. Following institutional review board approval, students’ unique identifiers were systematically uploaded to “Chongyixinli” -a secure adolescent psychological assessment platform developed by our research consortium. Prior to data collection, all survey administrators, school psychologists, and homeroom teachers completed standardized training modules on testing protocols and ethical guidelines. Participants completed assessments during scheduled computer laboratory sessions. Platform engineers and school IT specialists collaboratively provided technical support and emergency response throughout testing periods. On-site supervision was maintained by trained survey personnel and homeroom teachers to ensure procedural compliance and address participant inquiries. All data underwent encrypted transmission and were stored on password-protected secure cloud servers compliant with the General Data Protection Regulation.

The psychological screening protocol encompassed three key components: socio-demographic data collection, personality assessment, and SIB evaluation. Socio-demographic variables included sex, age (categorized into 16-18 years), family residence classified as urban or rural, and educational institution type, with participants stratified into three groups: high school students, vocational college attendees, and university undergraduates. Personality profiles were assessed through standardized questionnaires, while self-harm ideation and behaviors were systematically evaluated using validated psychometric instruments.

Psychoticism traits were assessed using the Psychoticism (P) subscale of EPQ-RSC, a 48-item instrument adapted for Chinese populations and comprised four subscales: Extraversion, Neuroticism, Psychoticism, and Lie scale (12 items each). T-scores were stratified as intermediate (43.3-56.7), high psychoticism tendency (HPT: 56.7-61.5), and typical high psychoticism (THP: >61.5), with established psychometric validity including internal consistency (α=0.54-0.60) and test-retest reliability (r= 0.67) for the P subscale. In this study, adolescents scoring >56.7 (> 70th percentile) on the P subscale were classified as having high psychoticism, further differentiated into HPT (56.7- 61.5) and THP (> 61.5) subgroups based on severity thresholds aligned with national normative data (20).

Self-harm ideation screening. The assessment employed a modified Chinese version of the Beck Suicide Ideation Scale (BSI-CV) (21), adapted by the Beijing Psychological Crisis Research and Intervention Center. This 4-item instrument evaluate suicidal/NSSI ideation severity through Likert-scale responses (0=never to 3=often), measuring: 1) life meaninglessness (past year), 2) passive death wishes (past year), 3) specific suicidal/NSSI ideation (past year), and 4) specific suicidal/NSSI ideation (past month). Validation in a Chongqing adolescent cohort (n=23,856) demonstrated strong internal consistency (Cronbach’s α=0.87) (22).

Self-harm behaviors screening. Self-harm behaviors were operationally defined as suicide attempts or non-suicidal self-injury (NSSI) and assessed using the “Suicide Attitude and Mental Health Status Questionnaire (College Version-IV)” (23), developed by the Beijing Psychological Crisis Research and Intervention Center. This instrument systematically evaluated behavioral characteristics, including presence, frequency, timing of the most recent episode, pre-/post-behavior medical consultations for psychological issues, treatment receipt, methods/tools employed, tool sources, motivations, and purposes. To determine the recency of self-harm acts, participants reporting prior behaviors were required to specify the exact date of their last episode, with these responses defining the analytical timeframe for “most recent” incidents. A confirmed positive case required: 1) endorsement of the screening item (“Have you ever engaged in self-harm behaviors such as taking medication overdoses or wrist cutting?”), 2) complete documentation of behavioral details, and 3) chronological validity verification to exclude incidents reported as occurring after assessment participation.

### 2.3 Statistical analysis

Statistical analyses were conducted using IBM SPSS Statistics 29.0 (Armonk, NY). Categorical variables were expressed as frequencies (percentages) to summarize demographic characteristics and SIB prevalence in adolescents with high psychoticism. Group comparisons between HPT and THP subgroups utilized chi-square tests or Fisher’s exact tests where appropriate, evaluating differences in sociodemographic profiles and SIB manifestations. Forward stepwise logistic regression analysis identified variables independently associated with SIB outcomes. Statistical significance was defined as two-tailed *p* < 0.05.

## 3 Results

### 3.1 Demographic disparities between adolescents with HPT and THP

From an initial cohort of 136,266 screened adolescents, 48,401 individuals aged 16-18 years were eligible for analysis. Within this age stratum, 6,027 participants (12.45%) met criteria for high psychoticism, comprising 2,391 (39.67%) with HPT and 3,636 (60.33%) with THP. The cohort demonstrated an age distribution of 2,128 (35.30%) 16-year-olds, 1,482 (24.60%) 17-year-olds, and 2,417 (40.10%) 18-year-olds. Female predominance was observed (65.4%, n=3,942), with 44.8% (n=2,699) residing in urban areas.

Comparative analysis revealed statistically significant disparities (*p*<0.05) between HPT and THP subgroups across three demographic parameters: sex distribution (χ²=126.074), age stratification (χ²=7.799), and school type (χ²=7.504). No significant intergroup difference emerged in family residence patterns. Full statistical details are presented in **Table 1**.

**Table 1:** Comparative demographic profiles of adolescents with high psychoticism: HPT vs. THP Subgroups in Chinese Adolescents (Aged 16-18 years; N=6,027).

Features		HPT	%	THP	%	*χ2*	*P*(two-side)
Sex	Male	1030	49.4	1055	50.6	126.074	<0.001
	Female	1361	34.5	2581	65.5		
Age	16	804	37.8	1324	62.2	7.799	0.020
	17	578	39.0	904	61.0		
	18	1009	41.7	1408	58.3		
Family residence	Urban	1077	39.9	1622	60.1	0.110	0.740
	Rural	1314	39.5	2014	60.5		
School type	University	674	41.3	958	58.7	7.504	0.023
	Vocational college	341	42.6	460	57.4		
	High school	1376	38.3	2218	61.7		

### 3.2 Analysis of self-harm ideation among adolescents with high psychoticism

Within the cohort of 6,027 adolescents with high psychoticism, 62.8% endorsed life meaninglessness, 47.2% reported passive death wishes, and 34.7% specific suicidal/NSSI ideation within the past year. Comparative data from age-matched general population peers (16–18 years) revealed significantly lower prevalence: 45.3% (life meaninglessness), 27.1% (passive death wishes), and 16.3% (specific suicidal/NSSI ideation).

**Figure 1:**
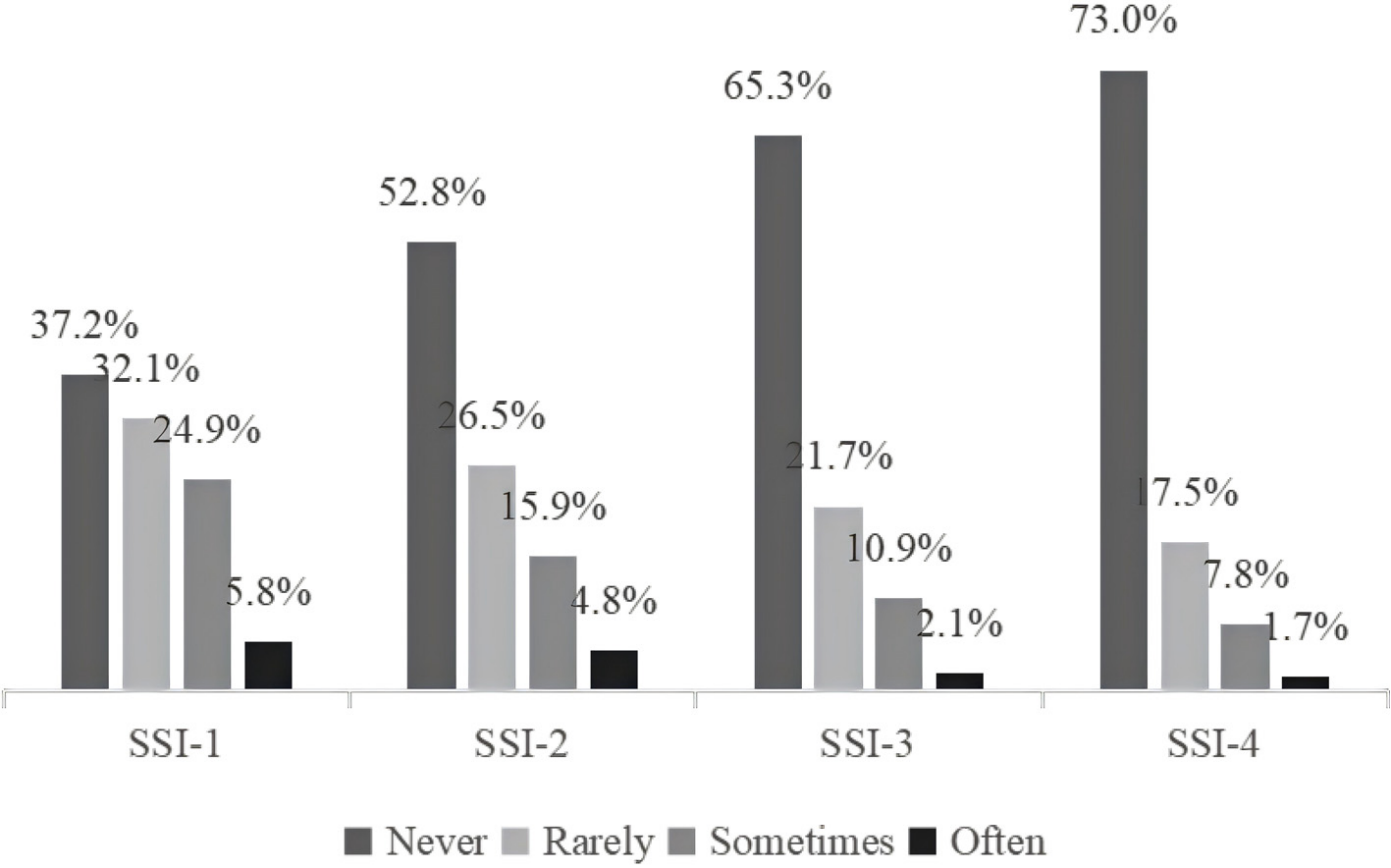
Proportions of self-harm ideation severity over the past year and past month in adolescents with high psychoticism (N=6,027). SSI-1= Life meaninglessness frequency (past year); SSI-2= Passive death wishes (past year); SSI-3= Specific suicidal/NSSI ideation (past year); SSI-4= Specific suicidal/NSSI ideation (past month).

Recent ideation patterns showed 27.0% of high-psychoticism adolescents reported specific suicidal/NSSI ideation in the past month, contrasting with 10.8% in the general adolescent population. After excluding duplicate responders, 8.0% exhibited frequent self-harm ideation past year, with 1.7% reporting persistent ideation past month (**Figure 1**).

**Figure 2:**
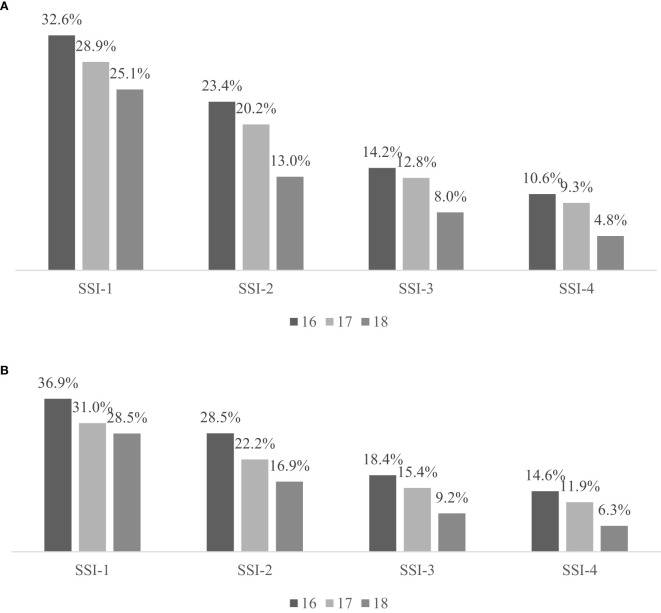
**(A)** Age-stratified prevalence estimates of high self-harm ideation among adolescents with HPT (n=2,391). **(B)** Age-stratified prevalence estimates of high self-harm ideation among adolescents with THP (n=3636). SSI-1= Life meaninglessness frequency (past year); SSI-2= Passive death wishes (past year); SSI-3= Specific suicidal/NSSI ideation (past year); SSI-4= Specific suicidal/NSSI ideation (past month).

Self-harm ideation frequency was operationalized through bimodal classification: “low ideation” (responses of never or rarely) versus “high ideation” (sometimes or often). Adolescents with high psychoticism demonstrated an age-dependent decline in high self-harm ideation prevalence, with distinct patterns observed between HPT and THP subgroups (**Figures 2A, B**).

### 3.3 Analysis of self-harm behaviors among adolescents with high psychoticism

**Figure 3:**
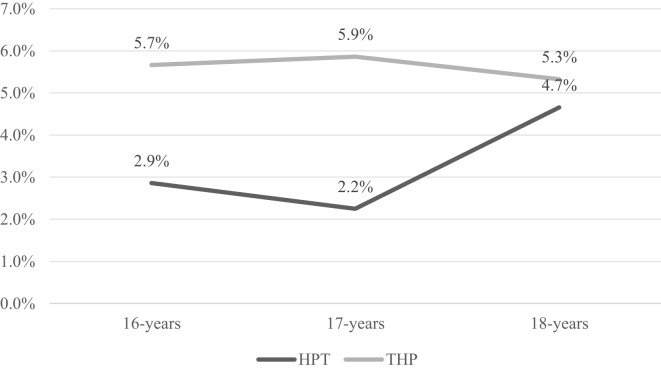
Age-stratified prevalence of lifetime self-harm behaviors in adolescents with HPT versus THP.

Lifetime prevalence of self-harm behaviors was substantially elevated in adolescents with high psychoticism (4.7%) compared to the general 16-18-year-old population (1.9%). Temporal patterns revealed the past-year self-harm behaviors prevalence of 1.64% and past-month prevalence of 0.37% within the high psychoticism cohort. Among those reporting lifetime self-harm behaviors, 59.8% exhibited recurrent episodes (≥2 occurrences). Comparative analysis demonstrated significantly higher self-harm behaviors rates in THP versus HPT subgroups across all ages, with both subgroups showing distinct inflection points at age 17 (**Figure 3**).

A detailed characterization of the most recent self-harm episode was conducted among adolescents with high psychoticism reporting such behaviors (n=286). Key parameters included temporal proximity of the event, pre-/post-episode healthcare-seeking behaviors for psychological distress, post-episode treatment engagement, method/tool utilization patterns, tool acquisition sources, behavioral reasons, and behavioral motivations. Clinical and contextual characteristics are systematically tabulated in **Table 2**.

**Table 2:** Clinical and contextual characteristics of the most recent self-harm episode among adolescents with high psychoticism (n=286).

Feature		n	%
Time of the most recent self-harm behavior	1 year before	165	57.7
	Past year	99	34.6
	Past month	22	7.7
Pre-/post-episode healthcare-seeking behaviors for psychological distress	Yes	28	9.8
	No	258	90.2
Post-episode treatment engagement	Yes	35	12.2
	No	251	87.8
Method/tool utilization patterns	Use of tools	171	59.8
	Taking poisons	31	10.8
	Taking therapeutic drugs	23	8.0
	Jumping	21	7.3
	Else	40	14.0
Tool acquisition sources	Putting at home	126	44.1
	Without tools	71	24.8
	Putting elsewhere	46	16.1
	Purchase specifically	43	15.0
Reasons for the behavior	Family conflicts	94	32.9
	Romantic issues	51	17.8
	Depression	49	17.1
	Work or academic problems	36	12.6
	Other mental health problem	23	8.0
	Interpersonal conflicts	17	5.9
	Else	10	3.5
	Related to ghosts and deities	3	1.0
	Financial difficulties	2	0.7
	Other financial difficulties	1	0.3
Behavioral motivations	Alleviate pain	146	51.0
	Evading responsibility	32	11.2
	Resisting reality	29	10.1
	Reducing the burden on others	24	8.4
	Else	21	7.3
	Retaliating against others	17	5.9
	Threatening others	17	5.9

### 3.4 Comparative analysis of SIB between HPT and THP subgroups

We performed comparative analyses of SIB between adolescents with HPT and THP, operationalizing SIB through five domains: 1) life meaninglessness (past year), 2) passive death wishes (past year), 3) specific suicidal/NSSI ideation (past year), 4) specific suicidal/NSSI ideation (past month), and 5) lifetime self-harm behaviors. Significant intergroup disparities were observed across all SIB dimensions, with detailed statistical outcomes presented in **Table 3**.

**Table 3:** Between-group comparisons of SIB patterns: HPT versus THP adolescents.

Feature		HPT	THP	χ2	P
Life meaninglessness (past year)	No	1709	2467	8.918	0.003
	Yes	682	1169		
Passive death wishes (past year)	No	1955	2819	15.708	<.001
	Yes	436	817		
Specific suicidal/NSSI ideation (past year)	No	2122	3123	10.437	0.001
	Yes	269	513		
Specific suicidal/NSSI ideation (past month)	No	2204	3247	13.818	<.001
	Yes	187	389		
Lifetime self-harm behaviors	No	2308	3433	14.230	<.001
	Yes	83	203		

### 3.5 Risk factor analysis for SIB in high psychoticism adolescents

Forward stepwise logistic regression models identified sex, psychoticism severity, and family residence as significant SIB predictors. Female adolescents exhibited 2.05-fold elevated SIB risk compared to males (95% CI 1.53-2.8). THP (vs. HPT) and rural residence conferred 1.51-fold (95% CI 1.16-1.96) and 1.32-fold (95% CI 1.04-1.68) risk increases, respectively.

The second multivariable-adjusted model incorporated lifetime self-harm behaviors, sex, psychoticism severity, family residence, and chronological age. Adolescents with lifetime self-harm behaviors demonstrated 7.19-fold elevated odds of past-year ideation compared to those without such history (95% CI 5.59-9.26). Each incremental year of age reduced ideation risk by 31.3% (aOR=0.687, 95% CI 0.627-0.753).

The third model adjusted for lifetime self-harm behaviors, age, sex, psychoticism severity, family residence, school type, and specific suicidal/NSSI ideation (past year). Those reporting past-year ideation exhibited 178.47-fold (95% CI 131.62-241.99) increased odds of specific suicidal/NSSI ideation (past month). Advancing age (aOR=0.767 per year, 95% CI 0.656-0.895) and female sex (aOR=0.648, 95% CI 0.486–0.864) (**Table 4**).

**Table 4:** Results of binary logistic regression analysis for SIB.

Feature	B	S.E.	Walt	P	OR	95%CI	
						Lower	Upper
Lifetime self-harm behaviors ^a^							
Family residence-Urban	0.278	0.122	5.200	0.023	1.320	1.040	1.675
Sex- Male	0.719	0.152	22.452	<.001	2.053	1.525	2.764
Degree of psychoticism- HPT	0.411	0.134	9.387	0.002	1.509	1.160	1.963
Specific suicidal/NSSI ideation (past year) ^b^							
Lifetime self-harm behaviors-No	1.973	0.129	235.302	<.001	7.194	5.590	9.256
Degree of psychoticism- HPT	0.176	0.083	4.499	0.034	1.192	1.013	1.402
Age	-0.375	0.047	64.147	<.001	0.687	0.627	0.753
Family residence-Urban	0.180	0.081	4.911	0.027	1.198	1.021	1.405
Specific suicidal/NSSI ideation (past year) ^c^							
Sex-Male	-0.434	0.147	8.718	0.003	0.648	0.486	0.864
Degree of psychoticism- HPT	0.365	0.144	6.391	0.011	1.440	1.085	1.911
Age	-0.266	0.079	11.283	<.001	0.767	0.656	0.895
Specific suicidal/NSSI ideation (past year)	5.184	0.155	1113.655	<.001	178.468	131.620	241.991

Forward LR binary regressive analysis was conducted. CI, Confidence Interval; OR, Odds Ratio; df, degree of freedom; S.E., Standard Error; HPT, High Psychoticism Tendency.

^a^Feature of “Sex” was input in step1, “degree of psychoticism” was input in step 2, and “Family residence” was input in step 3. Adjusting “Age” and “School type”.

^b^Feature of “Lifetime self-harm behaviors” was input in step1, “Age” was input in step 2, “Family residence” was input in step 3, and “Degree of psychoticism” was input in step 4. Adjusting “Sex”.

^c^Feature of “Specific suicidal/NSSI ideation (past year)” was input in step1, “Age” was input in step 2, “Sex” was input in step 3, and “Degree of psychoticism” was input in step 4. Adjusting “Lifetime self-harm behavior” and “Family residence”.

## 4 Discussion

To our knowledge, this study constitutes the first large-scale epidemiological investigation systematically characterizing SIB patterns and associated psychosocial determinants among adolescents with elevated psychoticism. By employing a robust sample and multi-dimensional assessment framework, we established critical associations between psychoticism severity gradients and SIB manifestation, while concurrently evaluating the modifying effects of socio-demographic covariates. These findings address a significant gap in developmental psychopathology literature by providing empirical evidence to inform the development of targeted SIB prevention protocols for this understudied high-risk population, ultimately contributing to the precision mental health interventions in adolescent populations.

### 4.1 Proportions and trends of SIB

Overall, adolescents with high psychoticism exhibited higher proportions of SIB compared to the general population. Chen et al. reported a 2.7% past-year suicide attempt rate among Chinese adolescents aged 9-18 (24), which exceeds our study’s finding of 1.64%. Notably, the lifetime prevalence of self-harm behaviors in high-psychoticism adolescents reached 4.7%, significantly surpassing the 1.9% observed in our general population sample of 16-18-year-olds. This rate also exceeded those reported in studies of adolescents under 18 years (3.5%) and first-year university students (1.2%) (7, 25). Our analysis identified 17 years as a critical developmental juncture, with both clinically significant and subthreshold psychoticism groups showing a turning point in self-harm behavior proportions. These findings align with previous research on suicide and NSSI among Chinese adolescents. Liu et al. documented that lifetime and past-year suicidal ideation, planning, and attempts in 12-18-year-olds had peaked at 16-17 years (26). The characteristic “inverted V” age distribution pattern, peaking at 15-16 years (27), suggests that maturing high-psychoticism adolescents may develop enhanced emotional regulation capacities, problem-solving skills, and cognitive integration, potentially explaining the subsequent decline in self-harm ideation. These patterns indicate that while self-harm behaviors in high-psychoticism adolescents share developmental trajectories with general populations, they manifest distinctive characteristics.

Despite demonstrating age-related declines in recent experiences of existential distress-with 62.8%, 47.2%, and 34.7% reporting thoughts of “life meaninglessness,” “death wishes,” and “suicidal/self-harm ideation” respectively- high psychoticism adolescents maintained substantially elevated rates compared to the general 16-18-year-old population. These proportions significantly exceeded those documented in Chinese college students, young adults, and general adolescent populations (7, 24, 25). Clinical analysis revealed a graded risk pattern: adolescents with THP showed 1.5, 1.2, and 1.4-fold increased risks for lifetime self-harm behaviors, past-year ideation, and past-month ideation respectively, compared to HPT. This dose-response relationship underscores psychoticism severity as a critical predictor of SIB risk in adolescents. Our findings substantiate the necessity of prioritizing high psychoticism youth in suicide prevention protocols and early intervention programs.

### 4.2 Characteristics of SIB

The predominant methods utilized by adolescents with high psychoticism traits in their most recent self-harm behaviors included tool-assisted injury, substance ingestion (encompassing toxic agents and therapeutic medications), and jumping from heights, aligning with previous reports on Chinese adolescent populations (23, 26). Enhanced regulation of accessible tools and medications in educational and domestic environments could effectively mitigate self-harm risks in this demographic. Notably, psychological pain relief constituted the primary motivation for self-harm behaviors in over 50% of cases. Defined as a complex state encompassing torment, loss, and intense negative affectivity that transcends clinical diagnostic boundaries (28), psychological pain demonstrates stronger predictive validity for suicidal tendencies than conventional indicators like depression (29). Recent investigations further substantiate that elevated psychoticism traits amplify the frequency of self-harm episodes through psychological pain mediation (5). The motivational-volitional model elucidates this phenomenon, positioning high psychoticism as a pre-motivational vulnerability factor (30). When confronted with familial conflicts, these adolescents frequently experience frustration-induced psychological distress. Their inherent impulsivity, risk-taking propensity, and underdeveloped problem-solving capacities collectively facilitate the transition from psychological distress to overt self-harm behavior manifestations.

Family conflicts emerged as the predominant precipitant of self-harm behaviors among adolescents with elevated psychoticism traits in our cohort (32.90% prevalence), nearly doubling the prevalence associated with romantic issues. This pattern contrasts with Chongqing collegiate populations where romantic difficulties constituted the primary trigger (26.04%) followed by familial discord (23.67%) (23). These findings highlight the disproportionate familial influence on adolescents with elevated psychoticism traits, necessitating prioritized family-centered interventions. Three-pronged therapeutic strategies were proposed: 1) Parental communication enhancement through school-family partnerships to cultivate supportive environments while improving caregivers’ mental health literacy; 2) Implementation of family therapy modalities emphasizing positive parenting practices to strengthen adolescent self-efficacy and psychological resilience; 3) Development of integrated surveillance systems combining household monitoring with comprehensive health education. Such multi-systemic approaches, synergizing familial, educational, and societal resources, may effectively mitigate SIB risk factors while promoting adolescent psychosocial well-being (31, 32).

Notably, while only 9% of adolescents with self-harm behaviors sought psychological consultation in our study, this represents a fifteen-fold increase from the 0.59% rate documented among Chongqing college students in 2008 (23). This upward trajectory correlates strongly with China’s evolving mental health policy framework over two decades. Initial milestones included the 1994 CPC Central Committee Guidelines mandating age-specific psychological education, followed by the 1995 Higher Education Moral Cultivation Outline formally incorporating mental health training (33). Subsequent regulatory evolution produced comprehensive frameworks including: the Adolescent Mental Health Promotion Initiative (2019-2022), 14th Five-Year Plan mental health provisions, and the 2023-2025 National Student Mental Health Enhancement Program (34–36). These policies mandated multi-system integration featuring: 1) School-based psychological platforms with staffing ratios of 1: 4,000 in universities and dedicated counselors in primary/secondary institutions; 2) Family engagement through parent-school collaborations reaching 50% of households; 3) Healthcare system capacity-building through specialized mental health infrastructure. Crucially, policy implementation monitoring through educational evaluation systems has operationalized these directives, collectively establishing China’s pioneering adolescent mental health protection network.

### 4.3 Determinants of self-harm

Our analysis identified rural family residence and elevated psychoticism levels as principal risk factors for SIB among adolescents with heightened psychoticism traits. While advancing age demonstrates protective effects against self-harm ideation, sex manifests differential risk patterns across temporal dimensions.

Sex-specific risk dynamics reveal longitudinal and cross-sectional disparities. Females exhibited a twofold higher lifetime SIB risk compared to males (*p<*0.001), potentially attributable to emotion regulation challenges that predispose adolescent girls to maladaptive coping strategies (37, 38). Paradoxically, recent data demonstrate a 35.2% lower 30-day self-harm ideation prevalence among females versus males (*p*=0.04), suggesting acute vulnerability in male adolescents. This temporal dichotomy necessitates sex-stratified prevention protocols: 1) For females, cognitive-behavioral interventions targeting emotional regulation skills; 2) For males, crisis-intervention systems addressing immediate stressors. Such tailored approaches optimize resource allocation while addressing distinct psychopathological mechanisms across gender groups.

Emerging evidence from depression research has empirically established psychoticism as a cardinal predictor of SIB, with paranoid ideation and depressive severity constituting principal predictors of SIB in major depressive disorder (7, 39). Depressed collegiate populations demonstrate significant psychoticism-SIB associations, where elevated psychoticism not only independently predicts suicidal ideation but also mediates ideation development through sequential cognitive pathways (40). Machine learning approaches further enable predictive modeling of NSSI in severely depressed students through psychoticism profiles combined with childhood trauma and social functioning metrics (41). Mechanistically, maladaptive coping modalities and affective dysregulation in high-psychoticism individuals may drive SIB via dual neurocognitive pathways: default mode network hyperconnectivity potentiates cognitive inflexibility during stress processing, while frontoparietal network hypoconnectivity compromises impulse regulation (42, 43). At the molecular level, NTRK2 gene polymorphisms (e.g., rs1360780) interact with psychoticism traits through prefrontal-limbic BDNF signaling dysregulation, as evidenced by multimodal neurogenetic studies (44). Epigenetic modulation via FKBP5 methylation downregulation reveals how childhood adversity induces persistent HPA axis dysregulation, potentially explaining familial conflict-triggered SIB escalation in psychoticism-prone adolescents (45). Future directions necessitate convergent multi-modal investigation combining real-time digital phenotyping (via wearable-based mood tracking), dynamic neural circuit mapping, and multi-omics profiling to elucidate the gene-brain-behavior continuum underlying psychoticism-related SIB pathogenesis.

This study has several notable strengths: firstly, it was based on a large-scale survey of adolescents with high psychoticism, providing data on age-related variations in self-harm ideation within this population. Secondly, the study identified significant differences in SIB between adolescents with HPT and THP. Additionally, it revealed the complex moderating role of sex in self-harm ideation among high psychoticism adolescents. Thirdly, the categorization of varying self-harm ideation severity facilitated cross-literature comparisons. However, limitations include the cross-sectional design precluding causal inference, and the lack of strict differentiation between suicide attempts and NSSI under the umbrella term “self-harm”, which may introduce comparative bias. Given the scarcity of SIB studies in this population, such comparisons remain contextually valuable. Furthermore, data collected from 2012–2015 (nearly a decade prior to analysis) may not reflect current SIB prevalence trends, potentially leading to overestimations or underestimations. However, our general 16-18-year-old cohort included high psychoticism adolescents without specific exclusion, which may affect prevalence estimates but enables broader comparisons. The lifetime prevalence of self-harm behaviors (1.9%) aligns with existing reports: intermediate between the 3.5% suicide attempt rate in Chinese adolescents <18 years (25) and 1.2% in Sichuan university freshmen (7). Similarly, the 16.3% past-year suicidal ideation proportion corresponds to the 15.2% reported in non-Western youth cohorts (46) from 1989–2018. While research on SIB risk factors in high psychoticism adolescents remains limited, our findings provide critical references. Future studies should prioritize updated data collection and refined behavioral distinctions to validate these observations.

In conclusion, adolescents exhibiting elevated psychoticism traits demonstrate a dose-dependent relationship between psychoticism severity and SIB risk, with significant demographic determinants including female sex, rural residence, and younger age. Preventive strategies should prioritize sex-specific interventions for female adolescents while implementing multi-system interventions encompassing evidence-based family education programs and accessible psychiatric services through community-based mental health infrastructures. These coordinated efforts aim to disrupt the psychopathological pathways linking psychoticism to SIB while addressing critical healthcare disparities in this vulnerable population.

## Data Availability

The original contributions presented in the study are included in the article/supplementary material. Further inquiries can be directed to the corresponding author.
